# Next generation L2-based HPV vaccines cross-protect against cutaneous papillomavirus infection and tumor development

**DOI:** 10.3389/fimmu.2022.1010790

**Published:** 2022-10-03

**Authors:** Melinda Ahmels, Filipe C. Mariz, Ilona Braspenning-Wesch, Sonja Stephan, Bettina Huber, Gabriele Schmidt, Rui Cao, Martin Müller, Reinhard Kirnbauer, Frank Rösl, Daniel Hasche

**Affiliations:** ^1^ Division of Viral Transformation Mechanisms, Research Program “Infection, Inflammation and Cancer”, German Cancer Research Center (DKFZ), Heidelberg, Germany; ^2^ Research Group Tumorvirus-specific Vaccination Strategies, Research Program “Infection, Inflammation and Cancer”, German Cancer Research Center (DKFZ), Heidelberg, Germany; ^3^ Laboratory of Viral Oncology, Department of Dermatology, Medical University of Vienna, Vienna, Austria; ^4^ Core Facility Unit Light Microscopy, German Cancer Research Center (DKFZ), Heidelberg, Germany

**Keywords:** cutaneous HPV, skin tumors, L2-based vaccine, next generation vaccine, animal model, cross-protection, *Mastomys coucha*, skin tumor formation

## Abstract

Licensed L1-VLP-based immunizations against high-risk mucosal human papillomavirus (HPV) types have been a great success in reducing anogenital cancers, although they are limited in their cross-protection against HPV types not covered by the vaccine. Further, their utility in protection against cutaneous HPV types, of which some contribute to non-melanoma skin cancer (NMSC) development, is rather low. Next generation vaccines achieve broadly cross-protective immunity against highly conserved sequences of L2. In this exploratory study, we tested two novel HPV vaccine candidates, HPV16 RG1-VLP and CUT-PANHPVAX, in the preclinical natural infection model *Mastomys coucha*. After immunization with either vaccines, a mock control or MnPV L1-VLPs, the animals were experimentally infected and monitored. Besides vaccine-specific seroconversion against HPV L2 peptides, the animals also developed cross-reactive antibodies against the cutaneous *Mastomys natalensis* papillomavirus (MnPV) L2, which were cross-neutralizing MnPV pseudovirions *in vitro*. Further, both L2-based vaccines also conferred *in vivo* protection as the viral loads in plucked hair after experimental infection were lower compared to mock-vaccinated control animals. Importantly, the formation of neutralizing antibodies, whether directed against L1-VLPs or L2, was able to prevent skin tumor formation and even microscopical signs of MnPV infection in the skin. For the first time, our study shows the proof-of-principle of next generation L2-based vaccines even across different PV genera in an infection animal model with its genuine PV. It provides fundamental insights into the humoral immunity elicited by L2-based vaccines against PV-induced skin tumors, with important implications to the design of next generation HPV vaccines.

## Introduction

Certain mucosal human papillomaviruses (HPV) are the etiological agents for several malignancies, including anogenital and head and neck cancer (1). Since HPV 16 and 18 are the most prevalent types, the first vaccines Cervarix^®^ and Gardasil^®^ were directed against these high-risk cancer-causing types. The latter additionally protects against HPV6 and 11, since these types, although considered as low-risk, can induce benign anogenital papillomas with high proliferation rates. Nowadays, a nonavalent vaccine is on the market (Gardasil^®^9), targeting the HPV types 6, 11, 16, 18, 31, 33, 45, 52 and 58, respectively (2, 3). All these vaccines are based on virus-like particles (VLPs), self-assembled from the major capsid protein L1, which are highly immunogenic and induce mostly type-restricted and high-titer neutralizing antibodies, but their potential for cross-protection is limited (2).

While mucosal HPVs are sexually transmitted and infection is age-dependent, cutaneous HPVs are part of the commensal skin microbiome that is passively acquired early after birth (4–6). While skin type HPV infection usually remains asymptomatic in healthy adults, numerous seroepidemiological and molecular studies showed that certain cutaneous HPV types are important co-factors in the development of non-melanoma skin cancer (NMSC) (7), the most frequent malignancy in the fair-skinned population (8). Elevated antibody titers against cutaneous HPVs as well as high viral loads in the skin correlate with an increased risk of developing squamous cell carcinomas (SCCs) (9–11).

Organ transplant recipients have an increased risk of developing NMSC (12, 13) and thus could especially benefit from a HPV vaccine targeting the plethora of betapapillomaviruses. Since no particular cutaneous HPV type predominates in NMSC (14, 15) there is a strong demand for broad-spectrum cutaneous HPV vaccines. In contrast to multivalent VLP vaccines licensed against anogenital disease, L2-based vaccines represent an alternative strategy. Here, the immune response is elicited by a stretch of amino acid residues (aa17-36 or aa20-38, respectively) at the N-terminus of the minor capsid protein L2, which is highly conserved among many HPV types (16, 17). In contrast to the type-restricted and high-titer neutralizing immune response induced by L1-VLP vaccination, immunization with the N-terminus of L2 (18, 19) can induce broadly cross-neutralizing yet low-titer antibodies against many mucosal and cutaneous HPVs. This led to the development of two L2-based vaccine candidates PANHPVAX (20) and HPV16 RG1-VLP (21), which are currently prepared for first-in-human clinical testing (clinical trial identifier: PANHPVAX: NCT05208710).

In the HPV16 RG1-VLP vaccine, the HPV16 RG1 epitope is genetically inserted into the immunogenic DE-surface loop of HPV16 L1 and displayed on the surface of assembled VLPs in a repetitive, closely spaced and highly immunogenic fashion (16, 21, 22), thus increasing the induction of long-lived antibody responses (23). As shown in heterologous preclinical models, vaccination induced broadly cross-neutralizing antibodies against high- and low-risk mucosal HPVs, some cutaneous HPVs and conferred *in vivo* cross-protection against all clinically relevant high-risk mucosal HPV types responsible for up to ~96% of all cervical cancers and several low-risk types. In addition, HPV16 RG1-VLP vaccinations induced a B cell memory and a vigorous cytotoxic T lymphocyte response (21).

The PANHPVAX consists of the L2 aa20-38 epitopes of eight mucosal HPV types that were grouped into multimeric polytopes (8mer) and inserted into the N-terminus of the thermo-resistant thioredoxin (Trx) scaffold protein of the archaea *Pyrococcus furiosus* (Pf) (20). This PfTrx-L2.8mer sequence was C-terminally fused to a hybrid derivative of the complement inhibitor C4-binding protein, referred to as OVX313 oligomerization domain (24). Recently, an updated version of this vaccine was designed by swapping the 8mer L2 polytope to aa20-38 epitopes of twelve cutaneous HPV types (c12mer). After expression of the PfTrx-L2.c12merOVX313 antigen in *E. coli* and thermal purification, immunization of mice and guinea pigs induces a broad immune response to various cutaneous HPV types which outperforms responses induced by the PANHPVAX (Mariz et al., 2022, in press). We refer to it as CUT-PANHPVAX.

However, protective capacity and efficacy of these vaccines to prevent viral infection *in vivo* can only be convincingly shown in a natural virus-host system and a final read-out in terms of tumor prevention. The African multimammate rodent *Mastomys coucha* is a unique model system to study natural infection with the cutaneous *Mastomys natalensis* papillomavirus (MnPV) in the context of skin carcinogenesis in an immunocompetent host (25). The animals almost entirely mimic the situation in humans in terms of onset of natural infection, viral persistence (26, 27) and cooperation with UV exposure during SCC development *via* a hit-and-run mechanism (28). Moreover, the availability of virus-free *Mastomys coucha* allows testing of different vaccination strategies *prior to* viral challenge at a defined time point under standardized conditions (29).

Here, we assessed the immunogenicity of two L2-based prophylactic HPV vaccines in *Mastomys coucha*. We demonstrate that the two vaccines elicit robust cross-reactive but distinct cross-neutralizing antibody responses against MnPV L2. Moreover, we observed that, when measurable, the cross-neutralizing antibody responses to the L2 protein induced upon vaccination are protective and prevent the development of MnPV-induced skin tumors. Interestingly, the immunity induced by the L2-based vaccines does not seem to be sterilizing, but strong enough to control virus load and prevent tumor development following infection.

## Results

### L2 vaccination - rationale and strategy

**Figure 1:**
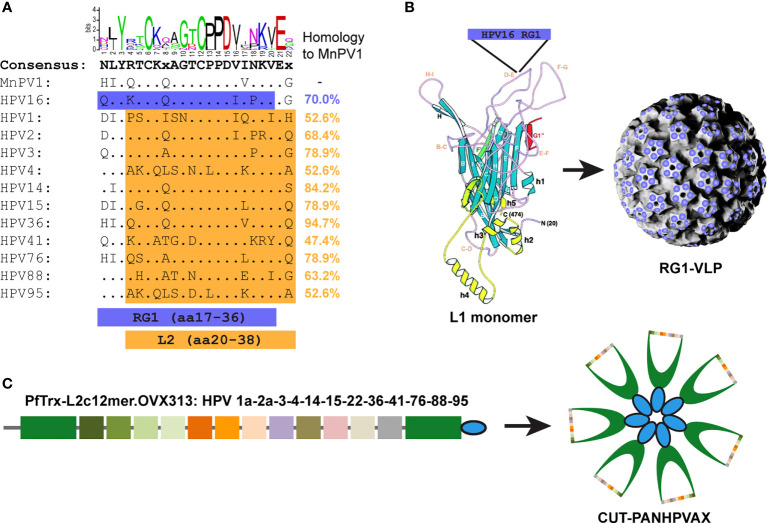
L2-based vaccines used in the study. **(A)** Alignment of L2 amino acid (aa) sequences of MnPV and HPV types either used in RG1-VLPs (HPV16 L2 aa17-36, referred to as ‘RG1’ epitope, blue) or CUT-PANHPVAX (L2 aa20-38, orange of indicated HPV types) vaccine. Sequence identities in comparison to MnPV L2 are expressed as percentage. The logos graphically depict the consensus sequence. **(B)** The RG1 epitope of HPV16 L2 is inserted into the DE surface loop of the HPV16 L1 monomer, creating a chimeric fusion protein. After self-assembly into complete VLPs, the RG1 epitope is exposed 360 times [modified from (30)]. **(C)** The CUT-PANHPVAX is a fusion protein consisting of L2 peptides aa20-38 derived from the cutaneous HPV types 1a, 2a, 3, 4, 14, 15, 22, 36, 41, 76, 88 and 95, respectively, inserted into the thioredoxin scaffold (PfTrx, shown in green) derived from the thermophile archaea *Pyrococcus furiosus*. The OVX313 domains (shown in blue) assemble to heat-stable heptamers, leading to a seven-fold presentation of the respective PfTrx-L2 in the PfTrx-L2-c12merOVX313 fusion protein (modified from Mariz et al., 2022, in press).

To examine the cross-protective capacity of two HPV L2-based vaccines in *Mastomys coucha*, virus-free animals were first immunized and subsequently experimentally infected with MnPV virions. **Figure 1A** shows the homology within the conserved L2 peptides that were used as antigens relative to MnPV L2. In the case of the HPV16 RG1-VLP vaccine (**Figure 1A**, marked in blue), where the L2 peptide is displayed 360x on the surface of completely assembled VLPs *via* the DE loop of L1 (**Figure 1B**), the sequence identity to MnPV L2 is 70% (18, 19). Alternatively, in the case of CUT-PANHPVAX (**Figure 1A**, orange), the sequence identity between MnPV L2 and the corresponding oligomerized L2 epitopes of different HPV types (**Figure 1C**) ranges between 47.4% and 94.7%. In both cases, the animal model *Mastomys coucha* will not only allow the examination of the B cell response, i.e. induction of neutralizing antibodies of the two vaccines, but will also provide evidence of whether MnPV-induced skin tumor formation can be prevented.

### Immunizations with HPV L2 vaccines induce cross-reactivity against MnPV L2

**Figure 2:**
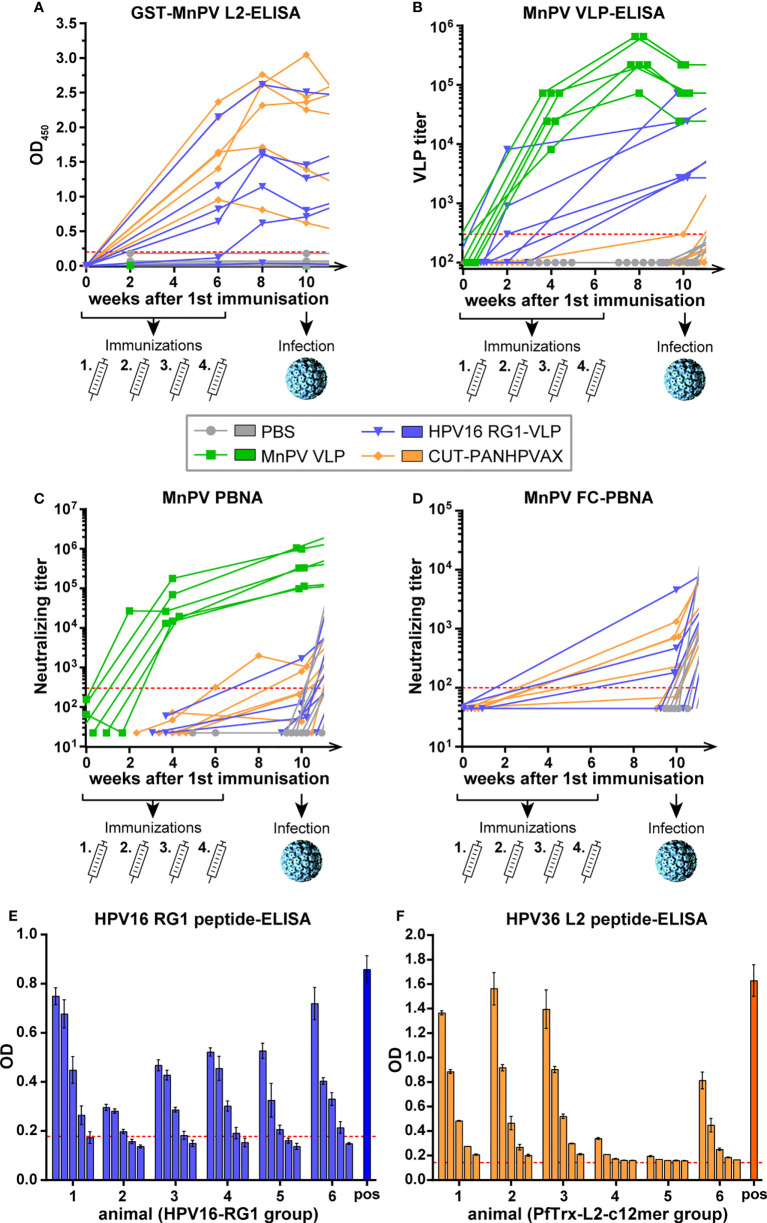
Monitoring seroconversion during vaccination. **(A)** GST-MnPV L2-ELISA. **(B)** MnPV VLP-ELISA and **(C)** MnPV PBNA and **(D)** MnPV FC-PBNA. The animals were vaccinated four times with MnPV VLPs (green), HPV16-RG1-VLPs (blue), CUT-PANHPVAX (orange) or injected with PBS (grey) in a bi-weekly interval from weeks 0 to 6 *prior to* an experimental MnPV infection at week 10. All groups consist of n=6 animals. Dashed lines represent the methods’ cut-off (OD_450_ = 0.2 for GST-ELISA; titer of 300 for VLP-ELISA and MnPV PBNA or titer of 100 for MnPV FC-PBNA) (Note that VLP sera were not measured in GST-MnPV L2-ELISA and the MnPV FC-PBNA due to limited amount of sera). **(E)** Sera from the HPV16 RG1-VLP group taken at week 10 were measured as triplicates in serial four-fold dilutions (starting at 1:100) in HPV16 RG1 peptide-ELISA. A HPV16 RG1-VLP raised rabbit serum was used as positive control. Error bars show the standard deviation. The dashed line represents the methods’ cut-off based on reactivity of PBS animals. **(F)** Sera from the CUT-PANHPVAX group taken at week 10 were measured as duplicates in serial three-fold dilutions (starting at 1:120) in HPV36 L2 peptide-ELISA. A previously characterized mouse serum raised against HPV38 L2 was used as positive control. Error bars show the standard deviation. The dashed line represents the methods’ cut-off based on reactivity of PBS animals.

To test the induction of (cross-)reactive antibodies against MnPV in an exploratory study, 8-week-old virus-free *Mastomys coucha* were vaccinated either with HPV16 RG1-VLPs, CUT-PANHPVAX, MnPV L1-VLPs (positive control) or PBS (negative control) using 6 animals per group (3 males, 3 females, respectively). Vaccinations were performed subcutaneously four times in bi-weekly intervals until week six with antigen/adjuvant combinations previously shown to have the best efficacy (31, 32), followed by an experimental infection with MnPV virions at week ten (outlined in **Figure 2**). During an observation period until week 62 (graphs continued in **Figure 4**), seroconversion against MnPV L2 and MnPV L1-VLPs was monitored by ELISA and pseudovirion-based neutralization assay (PBNA).

As demonstrated in **Figure 2A**, both mock- and VLP-vaccinated animals did not develop antibodies against MnPV L2 as tested in GST-ELISA (week 0 vs. week 10, ^ns^p>0.9999, Two-Way-ANOVA). Conversely, both HPV16 RG1-VLP- and CUT-PANHPVAX-vaccination increasingly cross-reacted against MnPV L2 which seems to be slightly stronger in some animals for the latter vaccine candidate (week 0 vs. week 10, ***p<0.0001, Two-Way-ANOVA) in comparison to HPV16 RG1-VLP (week 0 vs. week 10, **p=0.0065, Two-Way-ANOVA). This could be explained by an overall higher sequence identity of MnPV L2 with the respective c12mer epitopes when compared with the single RG1 epitope (see **Figure 1**). The presence of twelve distinct epitopes in the CUT-PANHPVAX vaccine might further increase the chance to induce cross-reactive antibodies rather than only one HPV16 RG1 epitope although repetitively displayed on HPV16 RG1-VLPs.

Consistent with previous results (29), sera from MnPV VLP-vaccinated animals showed strong reactivity in the MnPV VLP-ELISA (week 0 vs. week 10, ***p<0.0001, Two-Way-ANOVA) (**Figure 2B**). Moreover, sera obtained after HPV16 RG1-VLP-vaccination were also cross-reacting with MnPV VLPs, presumably indicating the presence of antibodies largely directed against L1-internal epitopes shared between HPV16 and MnPV. Although reactivity of all HPV16 RG1-VLP-vaccinated animals raised above the cut-off in the VLP-ELISA, this increase was statistically not significant (week 0 vs. week 10, ^ns^p=0.6930, Two-Way-ANOVA). Here, the small group size in this exploratory study has to be considered carefully and limits the power of all statistical analyses. All sera derived from CUT-PANHPVAX-vaccinated animals behaved similar to those of PBS controls and did not react in MnPV VLP-ELISA (week 0 vs. week 10, ^ns^p>0.9999, Two-Way-ANOVA).

In addition, sera were tested for (cross-)neutralization by PBNA using MnPV pseudovirions as previously shown (29) (**Figure 2C**). Again, MnPV VLP-vaccinated animals developed high titers of L1-mediated neutralization already after four weeks, that further increased by week ten (week 0 vs. week 10, ***p<0.0001, Two-Way-ANOVA). Conversely, vaccination either with HPV16 RG1-VLPs or CUT-PANHPVAX only showed weak cross-neutralization (both groups, week 0 vs. week 10, ^ns^p>0.9999, Two-Way-ANOVA) (**Figure 2C**), despite high anti-L2 antibody titers determined by GST-MnPV L2-ELISA (**Figure 2A**). This discrepancy might be due the low intrinsic sensitivity of the L1-based PBNA to detect L2-mediated neutralization (33).

To circumvent this experimental bias, we next tested the sera in a L2-specific furin-cleaved (FC-) PBNA by pretreating PsVs with furin to expose L2 and to render infectivity independent from initial L1-binding to heparan sulfate proteoglycans (HPSG) (34). This assay has a higher sensitivity for anti-L2-based neutralization and shows that 3 out of 6 HPV16 RG1-vaccinated animals and 4 out of 6 CUT-PANHPVAX-vaccinated animals developed MnPV L2 cross-neutralizing antibodies (**Figure 2D**).

In addition to the above mentioned results of MnPV-specific cross-reactivities induced by L2-based vaccinations, we next measured the specificity of HPV L2-raised seroreactivities in ELISAs using peptides corresponding to the respective vaccine. Using this approach, we confirmed that all animals specifically seroconverted against the HPV16 RG1 epitope of the RG1-VLP (**Figure 2E**) or against the HPV36 L2 peptide, which is part of the CUT-PANHPVAX vaccine (**Figure 2F**).

Notably, despite the fact that females and males used here were siblings of the same age, the males seemed to respond to a lesser degree when compared with the females. Here, due to the small group size (3 males and 3 females per group) of this pilot study, no conclusion can be drawn, but this observation should be considered in a larger study to avoid a sex-related bias (**Figure S1**).

### Correlations of ELISA reactivity with neutralizing activity in PBNA

**Figure 3:**
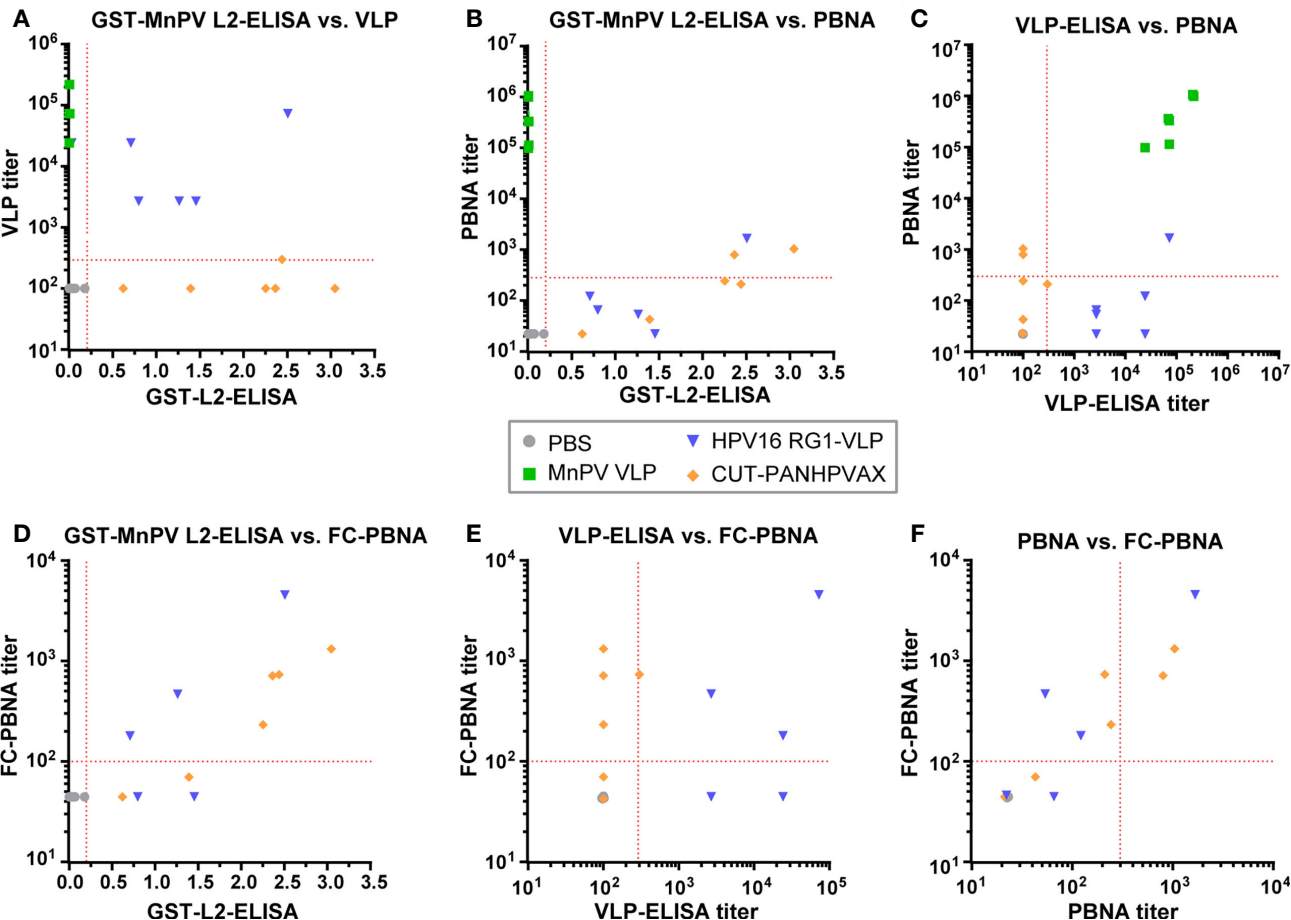
Correlation between GST-MnPV L2-ELISA, VLP-ELISA and PBNA data. Data at week 10 obtained from **(A)** GST-MnPV L2-ELISA and MnPV VLP-ELISA; **(B)** GST-MnPV L2-ELISA and MnPV PBNA; **(C)** MnPV VLP-ELISA and MnPV PBNA; **(D)** GST-MnPV L2-ELISA and FC-PBNA; **(E)** MnPV VLP-ELISA and FC-PBNA; **(F)** MnPV PBNA and FC-PBNA. Animals were vaccinated with MnPV VLPs (green squares), HPV16 RG1-VLPs (blue triangles), CUT-PANHPVAX (orange rhombi) or PBS (grey dots). All groups consist of n=6 animals. Dashed lines represent the methods’ cut-off (OD_450_ = 0.2 for GST-ELISA or titer of 300 for MnPV VLP-ELISA and MnPV PBNA or titer of 100 for MnPV FC-PBNA) (Note that sera of the MnPV VLP group were not measured in MnPV FC-PBNA due to limited amount of sera).

Next, seroreactivities were correlated with PBNA titers *prior to* viral infection (**Figure 3**). As shown in **Figure 3A**, after vaccination with CUT-PANHPVAX, the GST-MnPV L2-ELISA quite obviously did not correlate with the VLP-ELISA, exclusively measuring MnPV L1 antibodies. In contrast, elevated MnPV L2 cross-reactivity and VLP titers matched better for animals of the HPV16 RG1-VLP group. Nontheless, only strongly elevated MnPV L2 cross-reactivity correlated with cross-neutralization above the cut-off in the L1-PBNA (**Figure 3B**). Consequently, while titers in VLP-ELISA and L1-PBNA correlate well for VLP-vaccinated animals, this is not the case for the HPV16 RG1-VLP and CUT-PANHPVAX groups (**Figure 3C**). Both L2 vaccine groups have a much better correlation when L2-ELISA and FC-PBNA are compared (**Figure 3D**). Similar to the missing correlation with VLP-ELISAs (**Figure 3B**), GST-L2-seroreactivities of CUT-PANHPVAX-vaccinated animals did not correlate with L2-specific FC-PBNA and VLP-ELISA (**Figure 3E**) despite a related neutralizing efficacy in PBNA and FC-PBNA (**Figure 3F**). Sera of the HPV16 RG1-VLP group correlated better in all comparisons, but as mentioned above, additional antibodies were raised against non-neutralizing MnPV L1 epitopes, which may have affected the results of VLP-ELISAs and PBNAs. Independently of the L2-based vaccination, neutralization titers were relatively low (<10^4^, see **Figure 3D**), when compared to L1-based neutralization titers (10^5^-10^6^, see **Figure 3C**). This stresses the necessity of experimental systems that allow monitoring tumor incidences as a final read-out.

### Cross-reactive L2 antibodies are relatively stable over time

**Figure 4:**
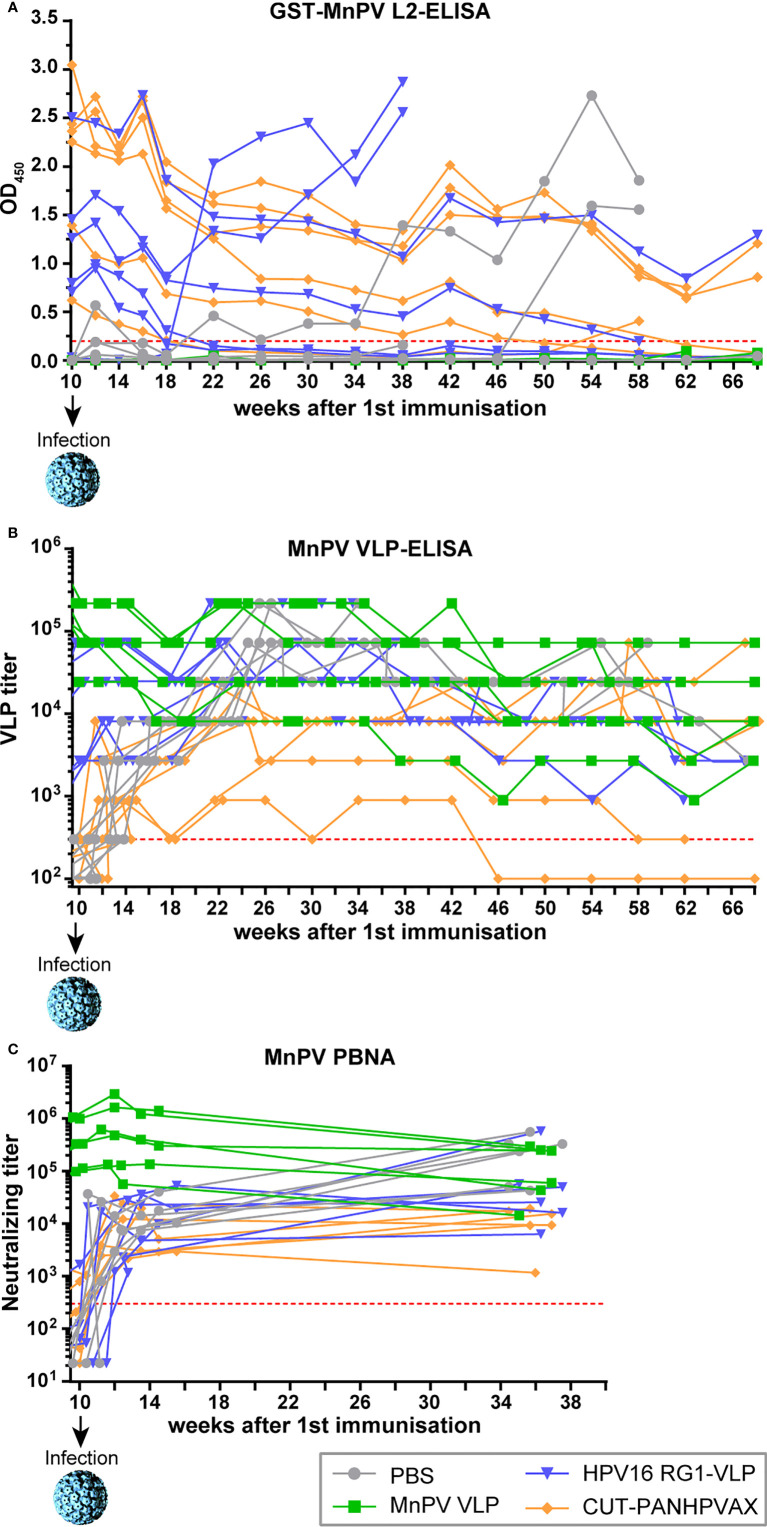
Monitoring of seroresponses of vaccinated animals after MnPV infection. At indicated time points, sera were measured by **(A)** GST-MnPV L2-ELISA, **(B)** MnPV VLP-ELISA and **(C)** MnPV PBNA. *Prior to* experimental infection at week 10, animals were vaccinated four times in bi-weekly intervals (see **Figure. 2**) with MnPV VLP (green), HPV16 RG1-VLP (blue), CUT-PANHPVAX (orange) or PBS (grey). All groups consist of n=6 animals. Dashed lines represent the methods’ cut-off (OD_450_ = 0.2 for GST-ELISA; titer of 300 for MnPV VLP-ELISA and MnPV PBNA).

Next, we monitored the course of the immune response of the different vaccines after viral challenge. For this purpose, four weeks after the last immunization, the animals were infected at their shaved backs with infectious MnPV virions obtained from a papilloma extract. At this time point, antibody titers had reached a stable plateau (**Figures 2A, B**). Reactivity against L2 was not dramatically increased in most animals in response to infection or even declined in some animals (**Figure 4A**), since L2 is hidden in the capsids of infectious virions and consequently does not represent an immunogenic structure *prior to* cleavage by furin. Conversely, as a result of infection, all animals except those of the MnPV VLP group strongly reacted against MnPV VLPs (**Figure 4B**) until week 14 and titers of neutralizing antibodies against MnPV raised clearly above the methods’ cut-off (**Figure 4C**). Since MnPV VLP-vaccinated animals had already developed high titers, experimental infection did not further boost their seroreactivity in VLP-ELISA and PBNA (**Figures 4B, C**).

Interestingly, in the two animals of the HPV16 RG1-VLP group that did not develop any cross-neutralizing antibodies measurable in MnPV L1-PBNA in response to vaccination, reactivity against MnPV L2 increased strongly after week 18 (**Figure 4A**). Therefore, despite the presence of cross-reactive antibodies against MnPV L2 in one of those two animals *prior to* infection and since both of them finally developed skin tumors, we considered them as non-responders. The same could be observed with two PBS controls after weeks 34 and 46, respectively, which developed tumors in the further course of the experiment. Notably, regardless of these four mentioned animals that strongly reacted in the MnPV L2-ELISA, seroreactivities of CUT-PANHPVAX- and HPV16 RG1-VLP-vaccinated animals declined until week 22 before reaching a plateau that remained relatively stable until week 46 before further declining (**Figure 4A**).

### Vaccination with HPV L2 vaccines cross-protects against MnPV infection and skin tumor formation *in vivo*

**Figure 5:**
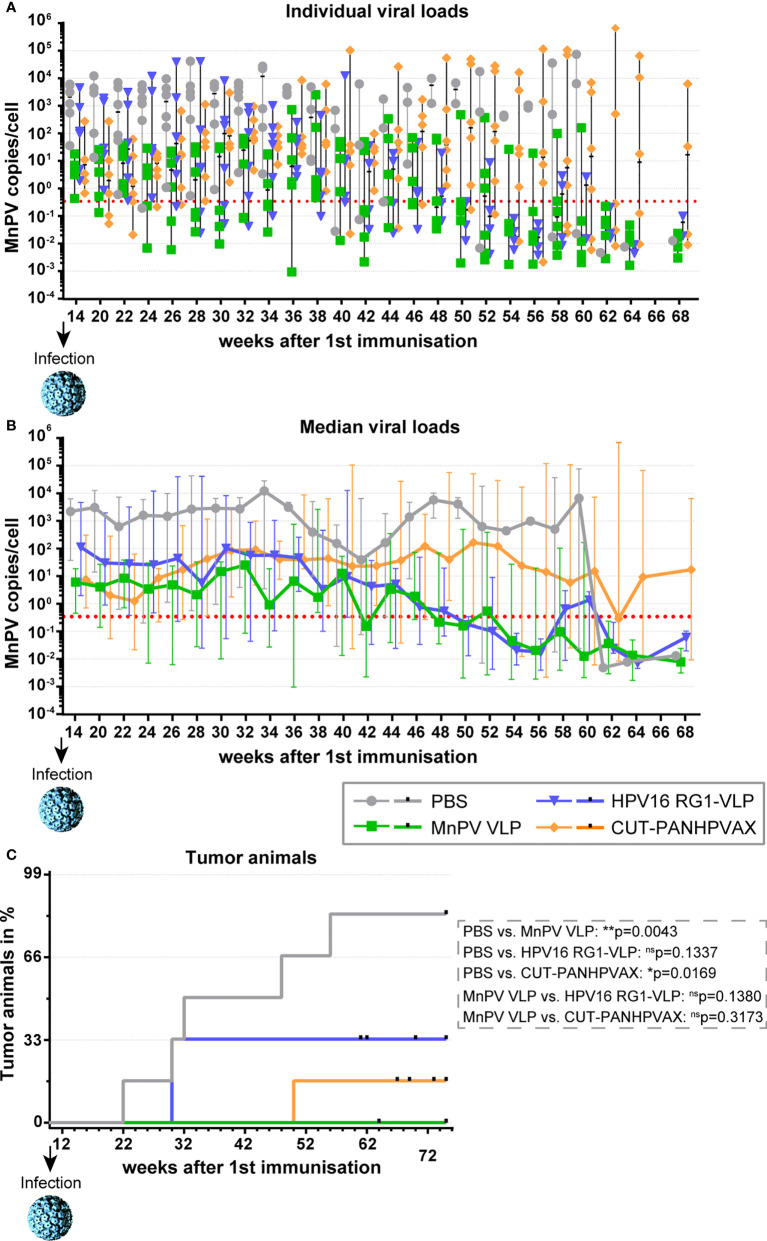
Monitoring viral load and tumor development after MnPV infection. **(A)** Viral load from plucked hair was followed over time *via* qPCR and is depicted **(A)** for individual animals or **(B)** for the vaccination groups (median ± range). Dashed lines represent the methods’ cut-off based on hair extracted *prior to* experimental infection (0.346 copies/cell). **(C)** Appearance of tumor-bearing animals in the different vaccination groups. Small vertical bars indicate censored animals which died for unknown reasons before tumor development. Differences between groups were calculated *via* log-Rank test.

To monitor whether the two HPV L2-based vaccines can cross-protect the animals against MnPV infection, the viral load in plucked hair bulbs was measured. As shown in **Figure 5A**, at week 14, the viral loads of all animals were above the methods’ cut-off, indicating that experimental infection was successful. Indeed, the median viral load of the PBS control group increased several log-folds (to 100-10,000 copies/cell) in comparison with the VLP group (1-10 copies/cell) (**Figure 5B**), clearly showing the protective effect of the vaccine. A median viral load comparable to the MnPV VLP group could be noted in both the CUT-PANHPVAX and HPV16 RG1-VLP group (fluctuating between 1-100 copies/cell). Here, the distribution in the HPV16 RG1-VLP group is broader than for the CUT-PANHPVAX, attributable to the statistical inclusion of the two vaccine non-responders that reached viral loads comparable to the PBS animals.

Consistent with their high viral load within the observation period of 75 weeks, five out of six (=83%) mock-vaccinated animals started developing visible skin tumors 12 weeks after experimental infection (**Figure 5C**). Conversely, all MnPV VLP-vaccinated animals remained tumor-free (p=0.0043; log-Rank test). In the CUT-PANHPVAX group, only one out of six (=17%) vaccinated animals developed a skin tumor at week 48 (PBS vs. CUT-PANHPVAX: p=0.0169; log-Rank test), which was accompanied by a high viral load. Considering HPV16 RG1-VLP-vaccinated animals, two out of six (=33%) also developed skin tumors, both at week 30 (PBS vs. HPV16 RG1-VLP: p=0.1337 log-Rank test). However, those two animals were the vaccine non-responders without cross-neutralizing seroreactivity in the MnPV L1-PBNA (both animals) and the MnPV L2-ELISA (one animal). Compared to the fully protective MnPV VLP-vaccination, both the HPV16 RG1-VLP and the CUT-PANHPVAX did not show significant differences in the protection efficacy (MnPV VLP vs. HPV16 RG1-VLP: p=0.1380, MnPV VLP vs. CUT-PANHPVAX: p=0.3173; log-Rank test).

**Figure 6:**
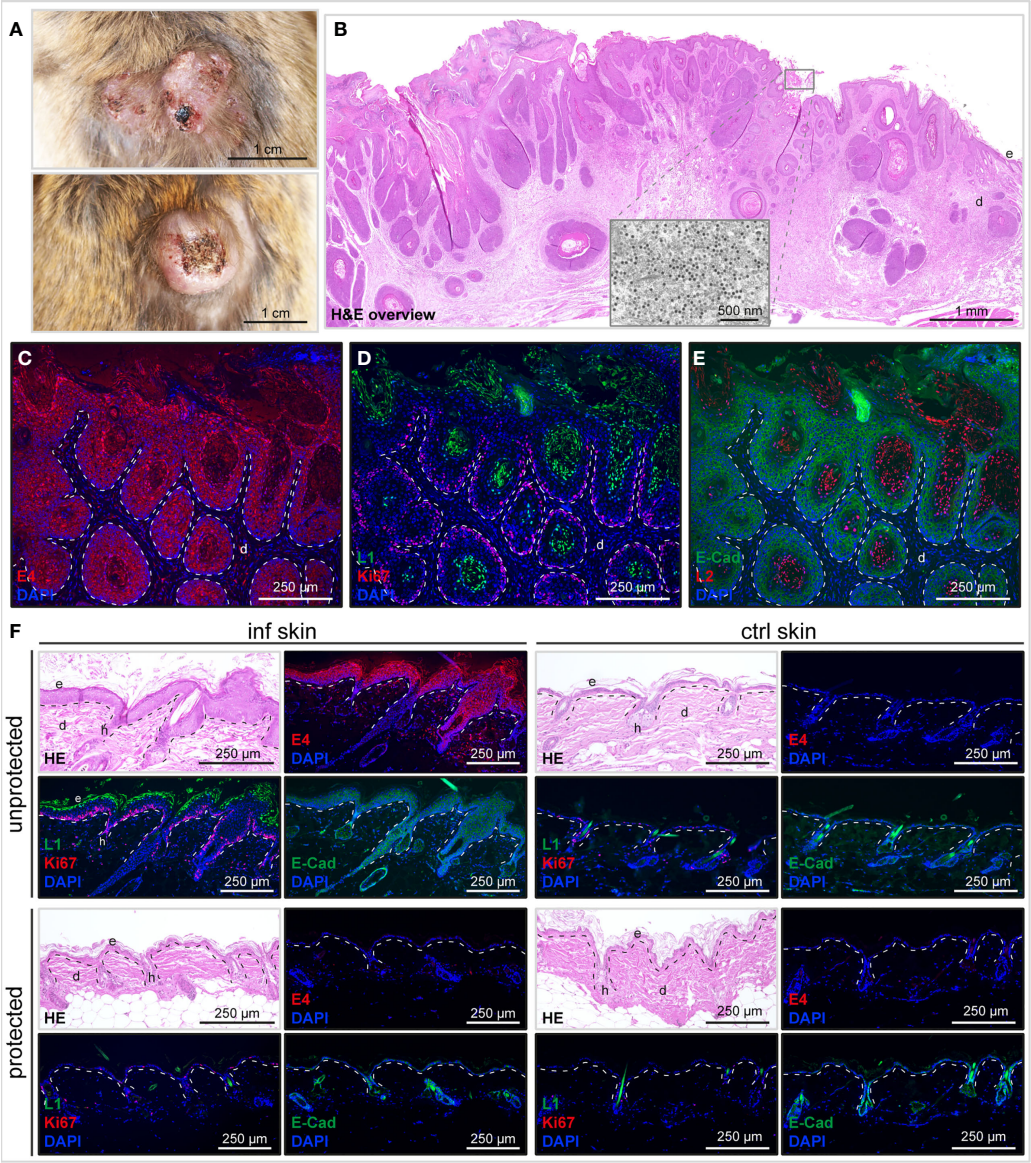
Histological characterization of MnPV-induced skin changes and tumor development. **(A)** Representative benign MnPV-induced skin tumors after experimental infection of unprotected animals. **(B)** HE staining of a benign MnPV-induced papilloma from the PBS control group. Virus progeny can be found in the keratinized outermost layer by transmission electron microscopy (TEM; inset). **(C)** Visualization of MnPV early gene expression by staining of MnPV E4 (red). **(D)** Proliferation rates are elevated in corresponding areas as indicated by Ki67 positive cells (red). Viral capsid components can be detected by staining of MnPV L1 (green) or **(E)** MnPV L2 (red). E-cadherin (green) staining was used to reveal the epidermal origin of infected cells. **(F)** Representative tissue stainings of unprotected and protected vaccinated animals. Experimentally infected (‘inf’) and uninfected control (‘ctrl’) skin of the same animals was Hematoxylin-Eosin (HE) stained or subjected to immunofluorescence (IF) for staining of MnPV E4 or Ki67 (both red), L1 or E-cadherin (both green). Nuclei were stained with DAPI (blue).

Skin lesions are initially macroscopically invisible and progress focally to larger palpable plaques that finally result in benign epithelial tumors (**Figures 6A, B**). Here, MnPV persists in high copy numbers and expresses both early (i.e. E4, protein, **Figure 6C**, red) and late (L1, **Figure 6D**, green; L2, **Figure 6E**, red), gene products, indicating that the viral permissive cycle is completed. Strong Ki67 staining (**Figure 6D**, red) and E-cadherin positivity (**Figure 6E**, green) reveals hyperproliferative MnPV-infected epidermal cells. Accordingly, such tumors represent a rich source of viral progeny (**Figure 6B**, inset) that is released by partially massive shedding. Abovementioned signs of infection can already be histologically observed in macroscopically inconspicuous skin (**Figure 6F**), but notably not in animals that had developed neutralizing antibodies upon vaccination.

Taken together, this pilot study shows that currently developed 2^nd^ generation HPV vaccines have indeed the potential to cross-protect against PV-induced epithelial hyperproliferation and skin tumor development even across different PV genera.

## Discussion

The aim of all vaccination strategies is to eradicate an infectious agent and associated diseases. Licensed multivalent L1-VLP-based vaccines against mucosal HPVs (35) are limited in their cross-protection against other high-risk HPV types (2). However, further increasing the number of VLP types has technical and economical limitations. Hence, alternative vaccine approaches currently utilize a highly conserved stretch of amino acids within the L2 protein as immunogen with the capacity to induce a broad-spectrum of cross-neutralization and cross-protection (36). The present study was designed to test two types of L2-based next generation vaccine candidates in a natural animal model (**Figure 1**): RG1-VLPs expose the HPV16 RG1 epitope on the surface of assembled VLPs (21, 22); CUT-PANHPVAX represents a vaccine that oligomerizes conserved L2 sequences of twelve different cutaneous HPV as a pan-specific antigen on a thioredoxin platform (24).

Their cross-protective efficacies were examined by vaccinating virus-free animals *prior to* experimental infection with MnPV virions and follow-up of seroconversion, viral load and tumor development over time. Designed as an exploratory study, MnPV VLPs were used as positive control since their protective effect could be previously shown even under immunosuppressive conditions (29). Besides inducing specific seroconversions against the peptides HPV16 RG1 (**Figure 2E**) or HPV36 L2 (**Figure 2F**) as a part of the CUT-PANHPVAX vaccine, both L2-based vaccines led to strong cross-reactivity against MnPV L2 (**Figure 2A**). This was mediated by aa17-38 within L2, a sequence that is well-conserved even amongst different genera of PVs (16). These antibodies also have cross-neutralizing potential as confirmed by FC-PBNA (**Figure 2D**), which is more sensitive to detect L2-based neutralization (**Figure 2C**) than the conventional PBNA (37, 38).

CUT-PANHPVAX vaccination seemed to exert a slightly better cross-reactive potential when compared with the HPV16 RG1-VLP, likely due to the presence of twelve distinct epitopes with partially high sequence identity to MnPV L2 (reaching 94.7%) (**Figure 1**). Conversely, the sequence identity between the RG1 peptides of the cutaneous MnPV and the mucosal HPV16 is only 70%. However, despite its 360-fold closely spaced reiteration on VLPs to increase immunogenicity (23, 39), this is still not high enough to induce robust cross-neutralization against MnPV. A potential advantageous side effect of the HPV16 RG1-VLP vaccination could theoretically be the additional induction of antibodies against L1 with a certain cross-neutralizing potential as described for conventional PBNAs (32, 40). Although sera of the HPV16 RG1-VLP-vaccinated group also reacted against MnPV VLPs (**Figure 2B**), cross-neutralization could only be observed for one animal when assessed by PBNA (**Figure 2C**). This indicates that cross-reactive anti-VLP antibodies are mainly induced by internal HPV16 VLP epitopes only accessible after accidental disruption of a minor fraction of the RG1-VLPs during antigen preparation and injection. Indeed, it was previously reported that cross-reactive antibodies are often directed against such linear epitopes, while (cross-)neutralizing antibodies are usually directed against conformation-dependent surface epitopes, comprised by different loops exposed on the capsids (41–43).

Regarding reactivity against L2, the differences of the vaccines have influenced their protection efficacy, since all six CUT-PANHPVAX-vaccinated animals developed antibodies cross-reactive against MnPV L2 and five of those also cross-neutralized furin-cleaved MnPV PsVs. However, after HPV16 RG1 vaccination, five out of six animals cross-reacted against MnPV L2 and only three were cross-neutralizing in FC-PBNAs (**Figure 2**).

Such an observation was recently reported for the nonavalent Gardasil^®^9 vaccine which could also not confer complete protection against some targeted HPV types - amongst them HPV16 - in a heterologous cutaneous rabbit challenge model (44). Therefore, it is not only necessary to find robust *in vitro* criteria but also adequate natural PV-infection models that allow a conclusive and reliable prediction of the *in vivo* protection efficacy of different HPV vaccines (45–47).

To assess the immediate effect of vaccination on the viral load after experimental infection, we quantified MnPV hair bulbs plucked from the back of the animals. For this purpose, we used a recently established chelex resin-based DNA extraction method followed by qPCR (48). As summarized in **Figures 5A, B**, vaccination with MnPV L1-VLPs efficiently kept the median viral load in a range of 1-10 copies/cell, which was up to ten times higher in the groups vaccinated with HPV16 RG1-VLPs or CUT-PANHPVAX. However, in mock vaccinated controls, a 100 to 1,000-fold higher viral load compared to L2-vaccinated mice was determined. This is consistent with the induction of high neutralizing antibody titers by vaccination with VLPs that interfere with cutaneous reinfection and viral spread. Mechanisms of neutralization include exudation or transudation of antibodies to the skin surface at sites of minor trauma or abrasion exposing the basement membrane, a requirement for preventing PV infection (2). The differences in median viral loads (**Figure 5B**) may result from different modes of action of L1- and L2-antibodies, since neutralization by L1-mediated opsonization happens as early as the virus reaches the epithelium. Conversely, anti-L2 antibodies only neutralize virions after they accessed the basement membrane and when further processed by furin convertase, which may allow a more narrowed time window for antibody binding. Therefore, L2-based immunization could induce a non-sterilizing humoral response different from that of VLP-based vaccines. Beyond blocking of infection, antibodies can also promote antiviral activity *via* recruitment and activation of innate immune cells and consequent induction of opsonophagocytosis (49). Importantly, these additional antibody-dependent responses are not measurable by the *in vitro* assays employed in this study, but may have contributed to the overall anti-viral effect induced by the L2-based vaccines in *Mastomys*.

Notably, several studies could correlate the amount of HPV DNA in skin to the risk of skin tumor development, for both healthy and immunocompromised individuals (50, 51). Of note, our animal model exactly mimics this scenario: unprotected mock-vaccinated animals accumulated high viral loads and developed skin tumors, while MnPV VLP-vaccinated animals completely remained tumor-free. Intriguingly, both outcomes could also be observed in the animals vaccinated with L2-based immunogens, correlating with the success of the vaccination (**Figure 5**). The results of this pilot study can be considered a proof-of-principle of a L2 vaccination-mediated *in vivo* protection.

Histological analyses revealed that in contrast to mock-vaccination and non-responders, skin of protected animals, irrespective of the induction mode of neutralizing antibodies (i.e. against L1-VLPs or L2-based antigens) did not develop thickened, hyperproliferative epidermis as premalignant signs of MnPV infection and eventually benign tumors (**Figure 6**). This is important, since in humans actinic keratoses (AK), considered as precursors of SCCs and associated with cutaneous HPV types (52), are already dermatologically treated to avoid malignant progression (53, 54). Considering a measurable viral load in hair bulbs despite induction of immunity by L2- or L1-based vaccines, this response does not appear to be completely sterilizing but strong enough to control viral load and prevent precursor lesions and tumor development after infection. In our model, strong keratinocyte proliferation in benign skin tumors is induced by MnPV to favor its own replication in parallel to host cell division. Substantial parakeratosis, accompanied by massive shedding represents a source of virion progeny. Their visualization by L1 and L2 stainings (**Figures 6E, F**) and in EM (**Figure 6C**) indicates that MnPV can complete its whole permissive cycle, which is absent in vaccinated animals.

In summary, this exploratory proof-of-concept study showed the protective efficacy of L2-based vaccines against MnPV challenge. It became obvious that animals with cross-neutralizing antibodies were protected against MnPV-induced skin tumors. However, statistical analyses should be considered with caution, as the statistical power was limited (6 animals per group). We clearly realized a statistically significant protective effect for the CUT-PANHPVAX vaccine when compared to the mock-vaccinated group (PBS, 5 of 6 animals vs. CUT-PANHPVAX, 1 of 6 animals: p=0.0169). In the case of the HPV16 RG1-VLP group that contained two non-responders that lacked neutralization in the MnPV L1-PBNA (both animals) and reactivity in the MnPV L2-ELISA (one animal) statistically one cannot conclude either an efficient or inefficient protection when compared to the PBS control (PBS, 5 of 6 animals vs. HPV16 RG1, 2 of 6 animals: p=0.1337) or the VLP-control (MnPV VLP, 0 of 6 animals vs. HPV16 RG1: p=0.1380). For the latter vaccine candidate a larger study would be needed to achieve more statistical power.

However, it is reasonable to assume that the HPV16 RG1-VLP vaccine in the form used here is limited in its efficacy to induce a broad cross-protection against cutaneous HPVs. The problem of low RG1 epitope sequence identity between mucosal and cutaneous HPV was already recognized and recently experimentally addressed by Olczak *et al.* (44), who combined the consensus RG1 epitope of betapapillomaviruses and the mucosal HPV16 RG1 in DE loops of VLPs, which revealed a higher potential to broadly cross-neutralize skin-specific PV types. Although optimizations are still needed, L2-based vaccination strategies have great, though not unlimited, potential to induce broad-based cross-protective immunity against cutaneous HPVs and related diseases. Their success in patients needs to be shown by clinical trials, some of which will start soon.

## Materials and methods

### Ethics statement

The animals are housed and handled in accordance with local (DKFZ), German and European statutes. All animal experiments were approved by responsible Animal Ethics Committee for the use and care of live animals (Regional Council of Karlsruhe, Germany, File No 35-9185.81/G289/15 and 35-9185.81/G65/21).

### Animals

Virus-free *Mastomys coucha* were obtained from Janvier Labs (Le Genest-Saint-Isle, France). At the DKFZ *Mastomys coucha* were housed under specified pathogen-free (SPF) conditions in individually ventilated cages (Tecniplast GR900) at 22+/-2˚C and 55+/-10% relative humidity in a light/dark cycle of 14/10 h. *Mastomys* were fed with mouse breeding diet and allowed access to water *ad libitum*. According to the three R rules of animal experimentation, the animals used in this exploratory study were subgroups (n=6 animals per group; groups: PBS, MnPV L1-VLPs, HPV16 RG1-VLPs, CUT-PANHPVAX) of a larger exploratory study also including testing the protective efficacy of an alternative L1 isoform which is published elsewhere (48).

### Antigen preparation

MnPV L1-VLPs were produced in and purified from Sf9 insect cells as recently described (26).

HPV16 RG1-VLPs were produced in *Spodoptera frugiperda* (Sf9) cells as described before (32, 55). RG1-VLPs were purified by ultracentrifugation on 35% (wt/vol) sucrose-PBS cushions and 29% (wt/wt) cesium chloride-PBS density gradients for 24 h prior followed by dialysis into 0.5M NaCl + 1mM CaCl_2_ + 0.01% Tween-80-PBS. Chimeric VLP purity and concentration was assessed by SDS-PAGE and Coomassie blue staining in reference to a Bovine Serum Albumin (Pierce) standard.

The PfTrx-L2c12mer antigen (referred to as CUT-PANHPVAX) was produced as described recently (Mariz et al., 2022, in press). Briefly, synthetic DNA encoding the antigen was cloned and expressed in *Escherichia coli* BL21 cells. The recombinant protein was purified by a heat-thermal purification step followed by ion-exchange chromatography. Prior to immunization, the protein sample was subjected to a detoxifying procedure with Triton X-114 to reduce levels of bacterial endotoxin below 8 IU/ml. Protein concentration and purity was monitored by SDS-PAGE-Coomassie blue staining and Bradford assay.

### Vaccination and experimental infection

Animals were immunized at an age of eight weeks and each group consisted of half males and females. MnPV L1-VLPs were dialyzed against 50 mM Hepes, 0.3 M NaCl, pH7.4 and 10 µg VLPs were prepared with PBS and 50% Sigma Adjuvant System (SAS) (Sigma-Aldrich, St. Louis, MO, USA), containing monophosphoryl lipid A (MPLA) and synthetic trehalose dicorynomycolate in squalene and Tween80 (29) as suggested by the manufacturer.

For immunization with HPV16 RG1-VLPs, 10 µg antigen were mixed with 500 µg Alhydrogel adjuvant 2% (InvivoGen, San Diego, CA, USA) and 50 µg MPLA (similar to the Cervarix adjuvant ASO4) and adjusted with PBS to 150 µl prior to incubation for 1 h at RT with a rotator (56).

For immunization with CUT-PANHPVAX, 20 µg antigen were prepared with PBS and 50% AddaVax (InvivoGen), a MF59-like squalene-based oil-in-water nano-emulsion, as suggested by the manufacturer (20) (Mariz et al., 2022, in press).

The PBS control group was injected with PBS and 50% AddaVax only.

For all antigens, a volume of 150 µl was injected subcutaneously in a skin fold of the neck.

Animals were vaccinated four times in a bi-weekly schedule and challenged two weeks later with MnPV. Experimental infection was performed at the shaved back of anaesthetized animals (3% isoflurane) that was superficially scratched six times longitudinally and six times transversally with tattoo needles prior to application of 30 µl extract of a MnPV-induced papilloma (containing infectious MnPV virions) that was obtained from a previous study (29).

Blood was taken in intervals from two to eight weeks by puncturing the submandibular vein of anaesthetized animals, starting at the age of eight weeks. For the follow-up experiment, animals were monitored for the duration of their lifetime until they had to be sacrificed due to tumor development or decrepitude.

### GST-capture ELISA

The ELISA was performed as recently described (26). Briefly, 96well PolySorb ELISA plates (Thermo Fisher Scientific, Rockford, IL, USA) were coated overnight at 4°C with glutathione-casein diluted in carbonate buffer (pH9.6). The next day, the plate was blocked for 1 h at 37°C with casein blocking buffer (CBB, 0.2% casein in PBST: 0.05% Tween-20 in PBS) and then incubated with a bacterial lysate containing the GST-MnPV-L2-SV40-tag fusion protein for 1 h. To remove unspecific reaction against bacterial proteins or the GST-SV40-tag fusion protein, *Mastomys* sera were diluted 1:50 in CBB containing GST-SV40-tag and pre-incubated for 1 h. ELISA plates were washed four times with PBST and pre-incubated sera was added. After 1 h, plates were washed four times and HRP-conjugated goat anti-mouse IgG (H+L) antibody (1:10,000 in CBB, 1:10,000 in CBB, Promega GmbH, Walldorf, Germany) was applied for 1 h. Antibodies were quantified colorimetrically by incubating with 100 μl/well substrate buffer for 8 min (0.1 mg/ml tetramethylbenzidine and 0.006% H_2_O_2_ in 100 mM sodium acetate, pH6.0). The enzymatic reaction was stopped with 50 μl/well 1 M sulfuric acid. The absorption was measured at 450 nm in a microplate reader (Labsystems Multiskan, Thermo Fisher Scientific, Rockford, IL, USA). To calculate the serum reactivity against the respective antigen, sera were tested in parallel against the GST-SV40-tag fusion protein and the reactivity was subtracted from the reactivity against the GST-MnPV-L2-SV40-tag. Each ELISA was performed in duplicates at least. The cut-offs were previously calculated individually for each antigen by measuring sera of virus-free animals and adding three standard deviations to the mean.

### VLP-ELISA

VLP-ELISAs were performed as previously described (29). Briefly, 96well PolySorb ELISA plates (Thermo Fisher Scientific, Rockford, IL, USA) were coated with 100 ng/well purified high quality MnPV L1-VLPs in 50 mM carbonate buffer pH9.6. The next day, plates were blocked with CBB and incubated for 1 h with three-fold serial dilutions (ranging from 1:100 to 1:656,100) of *Mastomys* sera in CBB. Then, plates were washed four times with PBST and incubated with goat anti-mouse IgG-HRP (1:10,000 in CBB, Promega GmbH, Walldorf, Germany). After four washes, color development and measurement was performed as described for the GST-ELISA. Antibody titer represents the last reciprocal serum dilution above the blank. The cut-off was set to a titer of 300 based on previous experiences with measuring sera of animals from virus-free and naturally MnPV-infected colonies (28).

### HPV36 L2 peptide-ELISA

Serocluster 96well “U” bottom plates (Costar, USA) were coated with 0.2 µg/well streptavidin (Sigma-Aldrich, Germany) overnight at 37°C. On the next day, plates were blocked with PBS (1.5% milk, 0.3% Tween) for 1 h at room temperature, and 0.03 µg of N-terminally biotinylated-HPV36 L2 peptide (GGSGQTCKQAGTCPPDVVNKVEQT) (GenScript Biotech, Netherlands) was added to the plates, which were incubated for another 1 h. Following a washing step with PBS (0.3% Tween), sera of animals immunized with the PfTrx-L2c12merOVX313 antigen (referred to as CUT-PANHPVAX) were serially diluted in PBS (1.5% milk, 0.3% Tween), according to a three-fold fashion (ranging from 1:120 to 1:29,160), and added in duplicate to the L2 peptide-containing plates, which were then incubated for 1 h at 37°C. Plates were then washed again, and anti-L2 antibody reactivity was revealed with HRP-conjugated goat-anti-mouse IgG (Southern Biotech, USA) diluted at 1:3,000 in blocking solution, following incubation for another 1 h at 37°C. After a further washing step, 100 μl of 2,2′-azino-bis(3-ethylbenz-thiazoline-6-sulfonic acid) (ABTS; 1 mg/ml in 100 mM sodium acetate-phosphate buffer, pH4.2, containing 0.015% H_2_O_2_) were added to each well and the colorimetric reaction was quantified at 405 nm with Multiskan Go (Thermo Fisher Scientific, USA) after 8 and 16 min. L2 antibody titers were determined by calculating the mean values of the duplicate, with the standard deviation shown as vertical bars. Assay cut-off was defined as the average absorbance observed across the unvaccinated animals. The positive control serum was obtained from a Balb/c mouse previously immunized with the PfTrx-L2c12merOVX313 (animal permit G248/16 (Regierungspräsidium Karlsruhe, according to the same vaccination schedule employed here for *M. coucha* in terms of age, dose and adjuvantation). The cut-off of 0.142 was calculated by measuring sera of virus-free animals and adding three standard deviations to the mean (0.117 + 0.025).

### HPV16 RG1 peptide-ELISA

Streptavidin plates (Thermo Fisher Scientific) were coated overnight at 4°C with 1 µg/well biotinylated RG1-peptide (JPT Peptide Technologies, Berlin, Germany) in coating buffer (0.1 M Tris/HCl pH7.4 + 0.15 M NaCl + 0.1% Tween-20). On the next day, plates were excessively washed with coating buffer and blocked with 1% milk/PBS for one hour. After washing, plates were incubated with four-fold serial serum dilutions (ranging from 1:100 to 1:102,400) for 1 h, washed again and incubated with an HRP-conjugated goat-anti mouse or anti-rabbit antibody (Bio-Rad) for 1 h. Plates were developed using ABTS (Roche) substrate and OD_405_ was determined (Opsys MR, Dynex Technologies). The cut-off of 0.177 was calculated by measuring sera of virus-free animals and adding three standard deviations to the mean (0.131 + 0.046).

### MnPV pseudovirion-based neutralization assay

As previously described (40), animal sera (tested in duplicates) were diluted in DMEM (Sigma-Aldrich, St. Louis, USA) with 10% FCS (Invitrogen, Carlsbad, CA, USA) and subjected to three-fold serial dilutions in 96well cell culture plates (Greiner Bio-One GmbH, Frickenhausen, Germany). Afterwards, 60 µl of diluted sera were mixed with 40 µl of pseudovirions (harboring a reporter plasmid encoding *Gaussia* luciferase) to final serum dilutions ranging from 1:100 to 1:1,968,300 and incubated for 15 min at RT. Then, 50 µl of 2.5×10^5^ HeLaT cells/ml in DMEM with 10% FCS were seeded onto the pseudovirion-serum mixture and cultured for 48 h at 37°C. The activity of secreted Gaussia luciferase was measured 15 min after adding coelenterazine substrate and Gaussia glow juice (PJK Biotech, Kleinblittersdorf, Germany) according to the manufacturer’s instructions in a microplate luminescence reader (Synergy 2, BioTek Instruments, Inc, Winooski, VT, USA). The neutralization titer represents the reciprocal of the highest dilution that reduces the signal by at least 50%. The cut-off was set to a titer of 300 based on previous experiences with measuring sera of animals from virus-free and naturally MnPV-infected colonies (28).

An alternative FC-PBNA that is better suited for the detection of L2 neutralizing antibodies and employed furin-cleaved (FC-)pseudovirions was performed as originally described (57). Differently from the standard PBNA, the neutralization of FC pseudovirions in the FC-PBNA is assessed in the furin-deficient LoVoT reporter cell line. Determination of neutralization titer followed the same principle described above.

### Determination of viral load

Hairs re-grew four weeks after experimental infection and were plucked bi-weekly with clean forceps, which yielded in approximately 100 hair roots from three random positions within the infected area for each time point. DNA was extracted *via* Chelex resin-based method where the hair was digested overnight in 150 µl Chelex resin (5% w/v in water; 100 - 200 mesh; Bio- Rad, Hercules, CA, USA) with 2 µg proteinase K in a ThermoMixer (Eppendorf, Hamburg, Germany) at 56°C and 300 rpm. The suspension was subsequently vortexed for 10 sec, heated at 99°C for exactly 8 min in a ThermoMixer (Eppendorf, Hamburg, Germany), vortexed again for 10 sec and centrifuged at room temperature for 3 min at 12,000×g to pellet the Chelex resin. The supernatant was transferred into a new tube and stored at 4°C (short-term) or -20°C (long-term).

The qPCR was performed with 1 µl DNA-containing supernatant from above per reaction using the iTaq Universal SYBR Green Supermix (Bio-Rad, Hercules, CA, USA), including 2.5 µg BSA (New England BioLabs, Frankfurt am Main, Germany) and forward/reverse primers for the MnPV L1 gene and the single-copy-number gene β-Globin to determine the number of input cell equivalents (29). Per reaction, MnPV DNA copy numbers were determined in duplicate by using standard curves generated in the same PCR run with a standard containing MnPV and β-globin plasmids. MnPV DNA load was defined as the number of MnPV genomes per two β-globin copies. The cut-off of 0.346 copies/cell was calculated by measuring hair samples from 50 animals of the virus-free colony and adding three standard deviations to the median (0.003 + 0.343).

### Tissue stainings

Staining of formalin-fixed, paraffin-embedded tumors was performed as previously described (28). Briefly, deparaffinized sections were subjected to heat-induced epitope retrieval (citrate buffer pH6.0), blocked with 5% goat serum and incubated with primary antibodies [a self-made mouse monoclonal anti-MnPV E4 antibody, a self-made *Mastomys* monoclonal anti-MnPV L1 antibody or a cross-reactive mouse monoclonal K18L2 antibody (18)] overnight at 4°C. After washing, slides were incubated with Alexa488- or Alexa594-conjugated anti-mouse antibodies (Invitrogen, Carlsbad, CA, USA) and nuclei were stained with DAPI. Sections were mounted with Dako Faramount Aqueous Mounting Medium (Dako North America, Inc, CA, USA) and imaged with a Keyence BZ-9000 Microscope (Keyence Deutschland GmbH, Neu-Isenburg, Germany) or a Hamamatsu NanoZoomer S60 (Hamamatsu, Hamamatsu City, Japan).

### Electron microscopy

L1 isoform preparations were fixed with buffered aldehyde solution (2% formaldehyde, 2% glutaraldehyde, 1 mM MgCl_2_, 2% sucrose in 100 mM calcium cacodylate, pH7.2), followed by post-fixation in buffered 1% OsO_4_, graded dehydration with ethanol and resin-embedding in epoxide (12 g glycid ether, 6.5 g NMA, 6.5 g DDSA, 400 μl DMP30; all from Serva, Heidelberg, Germany). Ultrathin sections at nominal thickness 60 nm and contrast-stained with lead-citrate and Uranylacetate were observed in a Zeiss EM 910 at 100 kV (Carl Zeiss, Oberkochen, Germany) and micrographs were taken with image-plates, scanned at 30 µm resolution (Ditabis micron, Pforzheim, Germany).

### Alignments

Papillomavirus L2 sequences were taken from PaVE (Papillomavirus Episteme; pave.niaid.nih.gov) (58) and aligned using Clustal 2.0.12. The consensus sequence was determined using EMBOSS Cons (https://www.ebi.ac.uk/Tools/msa/emboss_cons/) (59) and visualized with WebLogo (http://weblogo.berkeley.edu/) (60).

### Statistical analysis

Data analysis and graphical representation were done with GraphPad Prism 6.0 Software. Tumor development was calculated with the log-Rank test at 95% confidence interval and a p-value of 0.05 to assess significance.

## Data availability statement

The original contributions presented in the study are included in the article/**Supplementary Material**. Further inquiries can be directed to the corresponding authors.

## Ethics statement

The animal study was reviewed and approved by Animal Ethics Committee for the use and care of live animals (Regional Council of Karlsruhe, Germany, File No 35-9185.81/G289/15 and 35-9185.81/G65/21).

## Author contributions

Conceptualization: DH, FR, RK Methodology: DH, FR, FCM, RC, MM, RK Investigation: DH, SS, IB-W, MA, FCM, BH Data curation: DH, BH, FCM Formal analysis: DH, FCM, BH Visualization: DH Resources: FR, RC, GS, MM, RK Funding acquisition: DH, FR Project administration: DH Supervision: DH Writing – original draft: DH, FR, FCM, MM, RK. All authors contributed to the article and approved the submitted version.

## Funding

IB-W was funded by the Wilhelm Sander-Stiftung (grant number 2018.093.1 to DH). MA was funded by the German-Israeli Cooperation in Cancer Research (DKFZ-MOST), project number CA182 to FR. RC was supported by the China Scholarship Council (CSC) and Infect-ERA III, collaboration project HPV-MOTIVA (grant number 031L0095B to FR). FM was supported by the Baden-Württemberg Stiftung (Germany), project CHAnCE (project number WSF-030). The funders had no role in the data collection and analysis or preparation of the manuscript. BH was supported by a Hertha Firnberg fellowship of the Austrian Science Fund (FWF) (grant number T1078).

## Acknowledgments

We gratefully thank Dr. K. Richter (Central Unit Electron Microscopy, DKFZ) for acquisition of EM images. Support and assistance by the animal technicians and veterinarians of the Center for Preclinical Research at the DKFZ is also highly acknowledged. We thank Dr. T. Bund (DKFZ) for his support in digitalization of tumor sections. We thank Saeed Shafti-Keramat (Laboratory of Viral Oncology, Dermatology, MUV) for excellent technical assistance.

## Conflict of interest

RK is member of Pathovax LLC and its Scientific Advisory Board. Under a licensing agreement between PathoVax LLC and Medical University of Vienna, the University and Dr. Kirnbauer are entitled to royalties associated with an invention (RG1-VLP) described in this publication. These arrangements have been reviewed and approved by the Medical University of Vienna in accordance with its conflict-of-interest policies. MM is a co-inventor of PANHPVAX, a vaccine against low- and high-risk mucosal HPV types currently entering phase-I clinical trial. MM and FCM are co-inventors on a patent related to a cutaneous papillomavirus vaccine (WO2019063841).

The remaining authors declare that the research was conducted in the absence of any commercial or financial relationships that could be constructed as a potential conflict of interest.

## Publisher’s note

All claims expressed in this article are solely those of the authors and do not necessarily represent those of their affiliated organizations, or those of the publisher, the editors and the reviewers. Any product that may be evaluated in this article, or claim that may be made by its manufacturer, is not guaranteed or endorsed by the publisher.

## Supplementary material

Supplementary Figure 1 Comparison of seroreactivities of males and females in the HPV16 RG1-VLP and CUT-PANHPVAX groups. **(A)** Cross-reactivity in the GST-MnPV L2-ELISA, **(B)** cross-neutralization in the L1-PBNA and **(C)** cross-neutralization in the MnPV FC-PBNA (Note that the immune response of the MnPV VLP group was not measured in MnPV FC-PBNA due to limited amount of sera).

## References

[1] Gheit T. Mucosal and cutaneous human papillomavirus infections and cancer biology. Front Oncol (2019) 9:355. doi: 10.3389/fonc.2019.00355 31134154 PMC6517478

[2] Huber B Wang JW Roden RBS Kirnbauer R. RG1-VLP and other L2-based, broad-spectrum HPV vaccine candidates. J Clin Med (2021) 10(5):1044. doi: 10.3390/jcm10051044 33802456 PMC7959455

[3] Pogoda CS Roden RB Garcea RL. Immunizing against anogenital cancer: HPV vaccines. PloS Pathog (2016) 12(5):e1005587. doi: 10.1371/journal.ppat.1005587 27196109 PMC4873021

[4] Antonsson A Karanfilovska S Lindqvist PG Hansson BG. General acquisition of human papillomavirus infections of skin occurs in early infancy. J Clin Microbiol (2003) 41(6):2509–14. doi: 10.1128/JCM.41.6.2509-2514.2003 PMC156491 12791874

[5] Bouwes Bavinck JN Neale RE Abeni D Euvrard S Green AC Harwood CA. Multicenter study of the association between betapapillomavirus infection and cutaneous squamous cell carcinoma. Cancer Res (2010) 70(23):9777–86. doi: 10.1158/0008-5472.CAN-10-0352 21098702

[6] Weissenborn SJ De Koning MN Wieland U Quint WG Pfister HJ. Intrafamilial transmission and family-specific spectra of cutaneous betapapillomaviruses. J Virol (2009) 83(2):811–6. doi: 10.1128/JVI.01338-08 PMC2612409 18987132

[7] Hasche D Vinzón SE Rösl F. Cutaneous papillomaviruses and non-melanoma skin cancer: Causal agents or innocent bystanders? Front Microbiol (2018) 9:874. doi: 10.3389/fmicb.2018.00874 29770129 PMC5942179

[8] Lomas A Leonardi-Bee J Bath-Hextall F. A systematic review of worldwide incidence of nonmelanoma skin cancer. Br J Dermatol (2012) 166(5):1069–80. doi: 10.1111/j.1365-2133.2012.10830.x 22251204

[9] Genders RE Mazlom H Michel A Plasmeijer EI Quint KD Pawlita M. The presence of betapapillomavirus antibodies around transplantation predicts the development of keratinocyte carcinoma in organ transplant recipients: A cohort study. J Invest Dermatol (2015) 135(5):1275–82. doi: 10.1038/jid.2014.456 25347116

[10] Andersson K Michael KM Luostarinen T Waterboer T Gislefoss R Hakulinen T. Prospective study of human papillomavirus seropositivity and risk of nonmelanoma skin cancer. Am J Epidemiol (2012) 175(7):685–95. doi: 10.1093/aje/kwr373 22419740

[11] Neale RE Weissenborn S Abeni D Bavinck JN Euvrard S Feltkamp MC. Human papillomavirus load in eyebrow hair follicles and risk of cutaneous squamous cell carcinoma. Cancer Epidemiol Biomarkers Prev (2013) 22(4):719–27. doi: 10.1158/1055-9965.EPI-12-0917-T 23396961

[12] Euvrard S Kanitakis J Claudy A. Skin cancers after organ transplantation. N Engl J Med (2003) 348(17):1681–91. doi: 10.1056/NEJMra022137 12711744

[13] Ulrich C Kanitakis J Stockfleth E Euvrard S. Skin cancer in organ transplant recipients–where do we stand today? Am J Transplant (2008) 8(11):2192–8. doi: 10.1111/j.1600-6143.2008.02386.x 18782290

[14] Forslund O Iftner T Andersson K Lindelof B Hradil E Nordin P. Cutaneous human papillomaviruses found in sun-exposed skin: Beta-papillomavirus species 2 predominates in squamous cell carcinoma. J Infect Dis (2007) 196(6):876–83. doi: 10.1086/521031 PMC3795387 17703418

[15] Forslund O Antonsson A Nordin P Stenquist B Goran Hansson B. A broad range of human papillomavirus types detected with a general PCR method suitable for analysis of cutaneous tumours and normal skin. J Gen Virol (1999) 80(Pt 9):2437–43. doi: 10.1099/0022-1317-80-9-2437 10501499

[16] Gambhira R Karanam B Jagu S Roberts JN Buck CB Bossis I. A protective and broadly cross-neutralizing epitope of human papillomavirus L2. J Virol (2007) 81(24):13927–31. doi: 10.1128/JVI.00936-07 PMC2168823 17928339

[17] Rubio I Bolchi A Moretto N Canali E Gissmann L Tommasino M. Potent anti-HPV immune responses induced by tandem repeats of the HPV16 L2 (20 – 38) peptide displayed on bacterial thioredoxin. Vaccine (2009) 27(13):1949–56. doi: 10.1016/j.vaccine.2009.01.102 19368776

[18] Rubio I Seitz H Canali E Sehr P Bolchi A Tommasino M. The n-terminal region of the human papillomavirus L2 protein contains overlapping binding sites for neutralizing, cross-neutralizing and non-neutralizing antibodies. Virology (2011) 409(2):348–59. doi: 10.1016/j.virol.2010.10.017 21074234

[19] Schellenbacher C Roden RBS Kirnbauer R. Developments in L2-based human papillomavirus (HPV) vaccines. Virus Res (2017) 231:166–75. doi: 10.1016/j.virusres.2016.11.020 PMC5549463 27889616

[20] Pouyanfard S Spagnoli G Bulli L Balz K Yang F Odenwald C. Minor capsid protein L2 polytope induces broad protection against oncogenic and mucosal human papillomaviruses. J Virol (2018) 92(4):e01930-17. doi: 10.1128/JVI.01930-17 29212932 PMC5790957

[21] Schellenbacher C Kwak K Fink D Shafti-Keramat S Huber B Jindra C. Efficacy of RG1-VLP vaccination against infections with genital and cutaneous human papillomaviruses. J Invest Dermatol (2013) 133(12):2706–13. doi: 10.1038/jid.2013.253 PMC3826974 23752042

[22] Schellenbacher C Roden R Kirnbauer R. Chimeric L1-L2 virus-like particles as potential broad-spectrum human papillomavirus vaccines. J Virol (2009) 83(19):10085–95. doi: 10.1128/JVI.01088-09 PMC2748020 19640991

[23] Chackerian B Peabody DS. Factors that govern the induction of long-lived antibody responses. Viruses (2020) 12(1):74. doi: 10.3390/v12010074 PMC7019776 31936163

[24] Ogun SA Dumon-Seignovert L Marchand JB Holder AA Hill F. The oligomerization domain of C4-binding protein (C4bp) acts as an adjuvant, and the fusion protein comprised of the 19-kilodalton merozoite surface protein 1 fused with the murine C4bp domain protects mice against malaria. Infect Immun (2008) 76(8):3817–23. doi: 10.1128/IAI.01369-07 PMC2493234 18474650

[25] Hasche D Rösl F. Mastomys species as model systems for infectious diseases. Viruses (2019) 11(2):182. doi: 10.3390/v11020182 PMC6409723 30795569

[26] Fu Y Cao R Schäfer M Stephan S Braspenning-Wesch I Schmitt L. Expression of different L1 isoforms of mastomys natalensis papillomavirus as mechanism to circumvent adaptive immunity. Elife (2020) 9:e57626. doi: 10.7554/eLife.57626 32746966 PMC7402679

[27] Schäfer K Neumann J Waterboer T Rösl F. Serological markers for papillomavirus infection and skin tumour development in the rodent model mastomys coucha. J Gen Virol (2011) 92:383–94. doi: 10.1099/vir.0.023515-0 20965987

[28] Hasche D Stephan S Braspenning-Wesch I Mikulec J Niebler M Gröne HJ. The interplay of UV and cutaneous papillomavirus infection in skin cancer development. PloS Pathog (2017) 13(11):e1006723. doi: 10.1371/journal.ppat.1006723 29190285 PMC5708609

[29] Vinzón SE Braspenning-Wesch I Müller M Geissler EK Nindl I Gröne HJ. Protective vaccination against papillomavirus-induced skin tumors under immunocompetent and immunosuppressive conditions: A preclinical study using a natural outbred animal model. PloS Pathog (2014) 10(2):e1003924. doi: 10.1371/journal.ppat.1003924 24586150 PMC3930562

[30] Chen XS Garcea RL Goldberg I Casini G Harrison SC. Structure of small virus-like particles assembled from the L1 protein of human papillomavirus 16. Mol Cell (2000) 5(3):557–67. doi: 10.1016/s1097-2765(00)80449-9 10882140

[31] Spagnoli G Pouyanfard S Cavazzini D Canali E Maggi S Tommasino M. Broadly neutralizing antiviral responses induced by a single-molecule HPV vaccine based on thermostable thioredoxin-L2 multiepitope nanoparticles. Sci Rep (2017) 7(1):18000. doi: 10.1038/s41598-017-18177-1 29269879 PMC5740060

[32] Schellenbacher C Huber B Skoll M Shafti-Keramat S Roden RBS Kirnbauer R. Incorporation of RG1 epitope into HPV16L1-VLP does not compromise L1-specific immunity. Vaccine (2019) 37(27):3529–34. doi: 10.1016/j.vaccine.2019.05.011 PMC6832690 31147274

[33] Day PM Pang YY Kines RC Thompson CD Lowy DR Schiller JT. A human papillomavirus (HPV) *in vitro* neutralization assay that recapitulates the *in vitro* process of infection provides a sensitive measure of HPV L2 infection-inhibiting antibodies. Clin Vaccine Immunol (2012) 19(7):1075–82. doi: 10.1128/CVI.00139-12 PMC3393370 22593236

[34] Schiller JT Day PM Kines RC. Current understanding of the mechanism of HPV infection. Gynecol Oncol (2010) 118(1 Suppl):S12–7. doi: 10.1016/j.ygyno.2010.04.004 PMC3493113 20494219

[35] Gupta G Glueck R Patel PR. HPV vaccines: Global perspectives. Hum Vaccin Immunother (2017) 13(6):1–4. doi: 10.1080/21645515.2017.1289301 PMC5489288 28362244

[36] Olczak P Roden RBS. Progress in L2-based prophylactic vaccine development for protection against diverse human papillomavirus genotypes and associated diseases. Vaccines (Basel) (2020) 8(4):568. doi: 10.3390/vaccines8040568 PMC7712070 33019516

[37] Wang JW Jagu S Kwak K Wang C Peng S Kirnbauer R. Preparation and properties of a papillomavirus infectious intermediate and its utility for neutralization studies. Virology (2014) 449:304–16. doi: 10.1016/j.virol.2013.10.038 PMC3932537 24418565

[38] Wang JW Jagu S Wang C Kitchener HC Daayana S Stern PL. Measurement of neutralizing serum antibodies of patients vaccinated with human papillomavirus L1 or L2-based immunogens using furin-cleaved HPV pseudovirions. PloS One (2014) 9(7):e101576. doi: 10.1371/journal.pone.0101576 24999962 PMC4084990

[39] Yang R Murillo FM Delannoy MJ Blosser RL t. Yutzy WH Uematsu S. B lymphocyte activation by human papillomavirus-like particles directly induces ig class switch recombination *via* TLR4-MyD88. J Immunol (2005) 174(12):7912–9. doi: 10.4049/jimmunol.174.12.7912 15944297

[40] Buck CB Pastrana DV Lowy DR Schiller JT. Generation of HPV pseudovirions using transfection and their use in neutralization assays. Methods Mol Med (2005) 119:445–62. doi: 10.1385/1-59259-982-6:445 16350417

[41] Bissett SL Godi A Beddows S. The DE and FG loops of the HPV major capsid protein contribute to the epitopes of vaccine-induced cross-neutralising antibodies. Sci Rep (2016) 6:39730. doi: 10.1038/srep39730 28004837 PMC5177933

[42] Senger T Becker MR Schädlich L Waterboer T Gissmann L. Identification of b-cell epitopes on virus-like particles of cutaneous alpha-human papillomaviruses. J Virol (2009) 83(24):12692–701. doi: 10.1128/JVI.01582-09 PMC2786864 19793806

[43] Fleury MJ Touze A Alvarez E Carpentier G Clavel C Vautherot JF. Identification of type-specific and cross-reactive neutralizing conformational epitopes on the major capsid protein of human papillomavirus type 31. Arch Virol (2006) 151(8):1511–23. doi: 10.1007/s00705-006-0734-y PMC7086758 16508703

[44] Olczak P Matsui K Wong M Alvarez J Lambert P Christensen ND. RG2-VLP: a vaccine designed to broadly protect against anogenital and skin human papillomaviruses causing human cancer. J Virol (2022) 96(13):e0056622. doi: 10.1128/jvi.00566-22 35703545 PMC9278150

[45] Pattyn J Van Keer S Tjalma W Matheeussen V Van Damme P Vorsters A. Infection and vaccine-induced HPV-specific antibodies in cervicovaginal secretions. A Rev literature. Papillomavirus Res (2019) 8:100185. doi: 10.1016/j.pvr.2019.100185 PMC6804463 31494291

[46] Bachmann MF Kalinke U Althage A Freer G Burkhart C Roost H. The role of antibody concentration and avidity in antiviral protection. Science (1997) 276(5321):2024–7. doi: 10.1126/science.276.5321.2024 9197261

[47] Day PM Kines RC Thompson CD Jagu S Roden RB Lowy DR. *In vivo* mechanisms of vaccine-induced protection against HPV infection. Cell Host Microbe (2010) 8(3):260–70. doi: 10.1016/j.chom.2010.08.003 PMC2939057 20833377

[48] Hasche D Ahmels M Braspenning-Wesch I Stephan S Cao R Schmidt G. Isoforms of the papillomavirus major capsid protein differ in their ability to block viral spread and tumor formation. Front Immunol (2022) 13:811094. doi: 10.3389/fimmu.2022.811094 35359995 PMC8964102

[49] Wang JW Wu WH Huang TC Wong M Kwak K Ozato K. Roles of fc domain and exudation in L2 antibody-mediated protection against human papillomavirus. J Virol (2018) 92(15):e00572–18. doi: 10.1128/JVI.00572-18 29743371 PMC6052307

[50] Wieland U Kreuter A Pfister H. Human papillomavirus and immunosuppression. Curr Probl Dermatol (2014) 45:154–65. doi: 10.1159/000357907 24643184

[51] Wang J Aldabagh B Yu J Arron ST. Role of human papillomavirus in cutaneous squamous cell carcinoma: A meta-analysis. J Am Acad Dermatol (2014) 70(4):621–9. doi: 10.1016/j.jaad.2014.01.857 PMC3959664 24629358

[52] Mitsuishi T Kawana S Kato T Kawashima M. Human papillomavirus infection in actinic keratosis and bowen's disease: Comparative study with expression of cell-cycle regulatory proteins p21(Waf1/Cip1), p53, PCNA, ki-67, and bcl-2 in positive and negative lesions. Hum Pathol (2003) 34(9):886–92. doi: 10.1016/s0046-8177(03)00352-6 14562284

[53] de Oliveira ECV da Motta VRV Pantoja PC Ilha CSO Magalhaes RF Galadari H. Actinic keratosis - review for clinical practice. Int J Dermatol (2019) 58(4):400–7. doi: 10.1111/ijd.14147 30070357

[54] John SM Trakatelli M Gehring R Finlay K Fionda C Wittlich M. CONSENSUS REPORT: Recognizing non-melanoma skin cancer, including actinic keratosis, as an occupational disease - a call to action. J Eur Acad Dermatol Venereol (2016) 30 Suppl:3, 38–45. doi: 10.1111/jdv.13608 26995022

[55] Kirnbauer R Booy F Cheng N Lowy DR Schiller JT. Papillomavirus L1 major capsid protein self-assembles into virus-like particles that are highly immunogenic. Proc Natl Acad Sci U.S.A. (1992) 89(24):12180–4. doi: 10.1073/pnas.89.24.12180 PMC50722 1334560

[56] Huber B Schellenbacher C Jindra C Fink D Shafti-Keramat S Kirnbauer R. A chimeric 18L1-45RG1 virus-like particle vaccine cross-protects against oncogenic alpha-7 human papillomavirus types. PloS One (2015) 10(3):e0120152. doi: 10.1371/journal.pone.0120152 25790098 PMC4366228

[57] Wang JW Matsui K Pan Y Kwak K Peng S Kemp T. Production of furin-cleaved papillomavirus pseudovirions and their use for *In vitro* neutralization assays of L1- or L2-specific antibodies. Curr Protoc Microbiol (2015) 38:14B 5 1–26. doi: 10.1002/9780471729259.mc14b05s38 PMC4533841 26237105

[58] Van Doorslaer K Li Z Xirasagar S Maes P Kaminsky D Liou D. The papillomavirus episteme: A major update to the papillomavirus sequence database. Nucleic Acids Res (2017) 45(D1):D499–506. doi: 10.1093/nar/gkw879 PMC5210616 28053164

[59] Madeira F Pearce M Tivey ARN Basutkar P Lee J Edbali O. Search and sequence analysis tools services from EMBL-EBI in 2022. Nucleic Acids Res (2022) 50(W1):W276–W279. doi: 10.1093/nar/gkac240 PMC9252731 35412617

[60] Crooks GE Hon G Chandonia JM Brenner SE. WebLogo: A sequence logo generator. Genome Res (2004) 14(6):1188–90. doi: 10.1101/gr.849004 PMC419797 15173120

