# Supplementary figures and images for: Next generation L2-based HPV vaccines cross-protect against cutaneous papillomavirus infection and tumor development
Source: Front Immunol. 2022 Oct 3;13:1010790. doi: 10.3389/fimmu.2022.1010790 (PMC9574214; doi:10.3389/fimmu.2022.1010790)

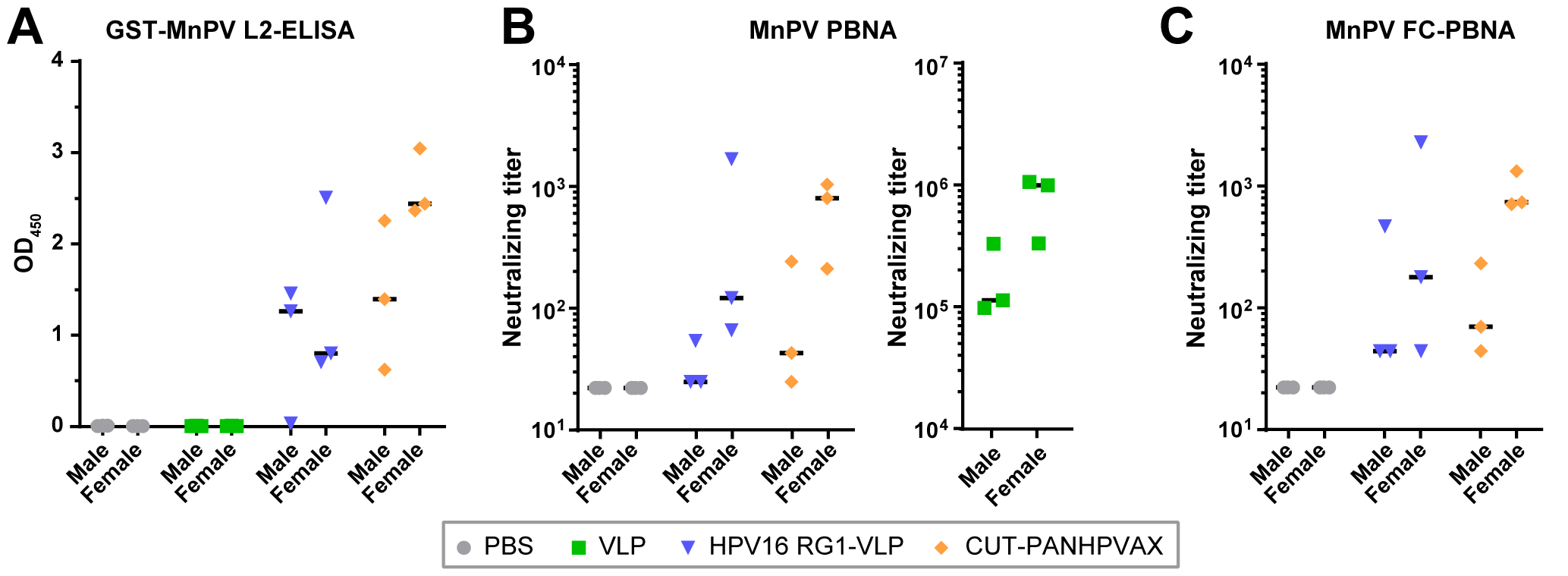

Supplement: Supplementary Figure 1 — Comparison of seroreactivities of males and females in the HPV16 RG1-VLP and CUT-PANHPVAX groups. (A) Cross-reactivity in the GST-MnPV L2-ELISA, (B) cross-neutralization in the L1-PBNA and (C) cross-neutralization in the MnPV FC-PBNA (Note that the immune response of the MnPV VLP group was not measured in MnPV FC-PBNA due to limited amount of sera). [file Image_1.tif]
